# The Na, K-ATPase β-Subunit Isoforms Expression in Glioblastoma Multiforme: Moonlighting Roles

**DOI:** 10.3390/ijms18112369

**Published:** 2017-11-08

**Authors:** Deborah Rotoli, Mariana-Mayela Cejas, María-del-Carmen Maeso, Natalia-Dolores Pérez-Rodríguez, Manuel Morales, Julio Ávila, Ali Mobasheri, Pablo Martín-Vasallo

**Affiliations:** 1 Laboratorio de Biología del Desarrollo, UD de Bioquímica y Biología Molecular and Centro de Investigaciones Biomédicas de Canarias (CIBICAN), Universidad de La Laguna, La Laguna, Av. Astrofísico Sánchez s/n, 38206 La Laguna, Tenerife, Spain; deborah_rotoli@yahoo.it (D.R.); mayela1050@gmail.com (M.-M.C.); javila@ull.es (J.Á.); 2 CNR–National Research Council, Institute of Endocrinology and Experimental Oncology (IEOS), Via Sergio Pansini, 5-80131 Naples, Italy; 3 Service of Pathology, University Hospital Nuestra Señora de Candelaria, 38010 Santa Cruz de Tenerife, Canary Islands, Spain; mmaefor@gmail.com; 4 Service of Medical Oncology, University Hospital Nuestra Señora de Candelaria, 38010 Santa Cruz de Tenerife, Canary Islands, Spain; natalia.perezrodriguez@gmail.com (N.-D.P.-R.); mmoraleg@ull.es (M.M.); 5 Medical Oncology, Hospiten® Hospitals, 38001 Santa Cruz de Tenerife, Tenerife, Spain; 6 Faculty of Health and Medical Sciences, University of Surrey, Guildford, Surrey GU2 7XH, UK; a.mobasheri@surrey.ac.uk

**Keywords:** Glioblastoma multiforme, Na, K-ATPase, sodium pump, Na, K-ATPase β subunit isoforms, moonlighting proteins, β2/AMOG, Glioblastoma multiforme microenvironment, astrocyte-neuron adhesion, Two-Hybrid system

## Abstract

Glioblastoma multiforme (GBM) is the most common form of malignant glioma. Recent studies point out that gliomas exploit ion channels and transporters, including Na, K-ATPase, to sustain their singular growth and invasion as they invade the brain parenchyma. Moreover, the different isoforms of the β-subunit of Na, K-ATPase have been implicated in regulating cellular dynamics, particularly during cancer progression. The aim of this study was to determine the Na, K-ATPase β subunit isoform subcellular expression patterns in all cell types responsible for microenvironment heterogeneity of GBM using immunohistochemical analysis. All three isoforms, β1, β2/AMOG (Adhesion Molecule On Glia) and β3, were found to be expressed in GBM samples. Generally, β1 isoform was not expressed by astrocytes, in both primary and secondary GBM, although other cell types (endothelial cells, pericytes, telocytes, macrophages) did express this isoform. β2/AMOG and β3 positive expression was observed in the cytoplasm, membrane and nuclear envelope of astrocytes and GFAP (Glial Fibrillary Acidic Protein) negative cells. Interestingly, differences in isoforms expression have been observed between primary and secondary GBM: in secondary GBM, β2 isoform expression in astrocytes was lower than that observed in primary GBM, while the expression of the β3 subunit was more intense. These changes in β subunit isoforms expression in GBM could be related to a different ionic handling, to a different relationship between astrocyte and neuron (β2/AMOG) and to changes in the moonlighting roles of Na, K-ATPase β subunits as adaptor proteins and transcription factors.

## 1. Introduction

Glioblastoma multiforme (GBM) is the most aggressive of malignant glioma. Even after state-of-the art treatment, the median survival of patients is less than one year and outcomes give overall survival (OS) as less than 10% at two years, decreasing to less than 2% at five years [1,2,3]. Heterogeneity of cells in GBM is a key factor for the low effectiveness of treatments [4]. GBM presents epigenetically and genetically different cell sub-populations within a single tumor that contributes to growth, progression and treatment failure. In fact, the term “multiforme” describes its heterogeneous histopathological features [5]. Primary GBM arises suddenly in older patients after a brief clinical history and is characterized by rapid progression and short survival time [6]. Secondary GBM are more frequent in younger patients and evolve from a diffuse or an anaplastic astrocytoma [7]. GBM consists of the following cell types: glioma stem cells (GSCs), astrocytes, vascular cells (endothelial and pericytes) [8,9], telocytes (a characteristic type of stromal cell, with thin prolongations up to hundreds of microns, ranging from the optic to electronic microscopy resolving power [10,11]), immune cells (glioma-infiltrating myeloid cells (GIMs) or tumor-associated macrophages (TAMs), and remaining neurons.

Despite the cellular heterogeneity in GBM [9,12], there are cellular processes and gene families that are common to every GBM cell and these could be used as probes for gaining a better understanding of GBM biology, clinical prognosis and response to therapy. Recent studies point out that gliomas exploit ion channels and transporters, including Na, K-ATPase, to sustain their singular growth and invasion as they invade the brain parenchyma [13]. The interest in Na, K-ATPase in brain tumors appeared soon after its discovery [14] and a decrease in its activity in gliomas was a striking finding. However, since then further research on this topic in GBM has been limited.

Na, K-ATPase is a plasma membrane embedded protein in all animal cells. Through the hydrolysis of an ATP molecule it transports three sodium ions out and two potassium ions into the cell, against steep electrochemical gradients [15]. This system regulates the cellular ionic homeostasis and maintains the electrochemical gradients required for ion channel function and secondary active transport [16]. Besides this, Na, K-ATPase is the receptor of cardiotonic glycosides. Recently, additional functions for Na, K-ATPase in the cell have been proposed. Na, K-ATPase is a signal transducer and transcription activator [17,18,19,20,21] affecting cell proliferation [22], and is involved in cell motility [23], and apoptosis [24]. A recent review describes the molecular basis of Na, K-ATPase involvement in cell proliferation and hypertrophy, apoptosis, cell adhesion, cell migration, signal transduction pathways and sodium pump-binding drugs [25].

A functional pump is composed of a catalytic α (100–112 kDa), a regulatory β (45–55 kDa) subunit and an optional γ (FXYD2) (6.5–10 kDa) subunit [26]. The Na, K-ATPase multigene family is constituted by several isoforms. Four different members of the α subunit have been found in humans [27]. FXYD contains at least seven isoforms in mammals [28]. Three different isoforms have been identified of the β subunit: β1 (ATP1B1 gene), β2 (ATP1B2 gene) and β3 (ATP1B3 gene) [16,29]. All isoforms associate promiscuously to create a functional pump. Furthermore, β2 is an adhesion molecule on glia (AMOG) involved in molecular interactions between neurons and glia [30].

Cardiotonic steroids, such as the hemisynthetic derivative of 2″-oxovoruscharin (UNBS1450), have been proposed for the treatment of GBM patients who do not respond to chemotherapy and whose tumors over-express the α1 isoform [31,32,33]. Furthermore, the effect of perillyl alcohol on the Na, K-ATPase appears to be the basis for arresting cell migration and activating pro-apoptotic pathways in human and murine glioma cell lines as well as in explanted tumor cells from a glioblastoma patient [34].

Na, K-ATPase β1 subunit isoform is expressed in almost all tissues and cells, while the expression of the other β isoforms is more restricted to certain tissues and cells. The β2 isoform [35] is found in skeletal muscle [36], and nervous tissues [37], whereas β3 is present in the testis, retina, optic nerve, corpus callosum, dorsal root ganglia, sciatic nerve, liver, and lung [38,39,40,41]. Astrocytes express β1 and β2 isoforms [37,42,43,44,45], although β2 predominates when the cells are fully differentiated [46,47]. C6-glioblastoma cells showed expression of only the β3 isoforms [40].

The β-subunits of Na, K-ATPase have been implicated in regulating cellular adhesion, particularly during cancer progression [48,49,50], and several laboratories have shown differential expression, altered subcellular localization and down regulation of the β subunits of the Na, K-ATPase in carcinoma cells [51,52,53,54]. β2/AMOG isoform has been implicated in the oncobiology of GBM [55,56,57].

Specific antisera against cell- and function-specific markers (Table 1) allowed us to study the involvement of β Na, K-ATPase subunit isoforms in the oncobiology and microenvironmental heterogeneity of primary and secondary GBM. Specific immunoreactivity is present in virtually all GBM cell kinds, showing a unique expression phenotype of β isoforms involved in the pathogenesis and progression of GBM.

## 2. Results

### 2.1. Na, K-ATPase β1 Isoform Expression in GBM

**Figure 1:**
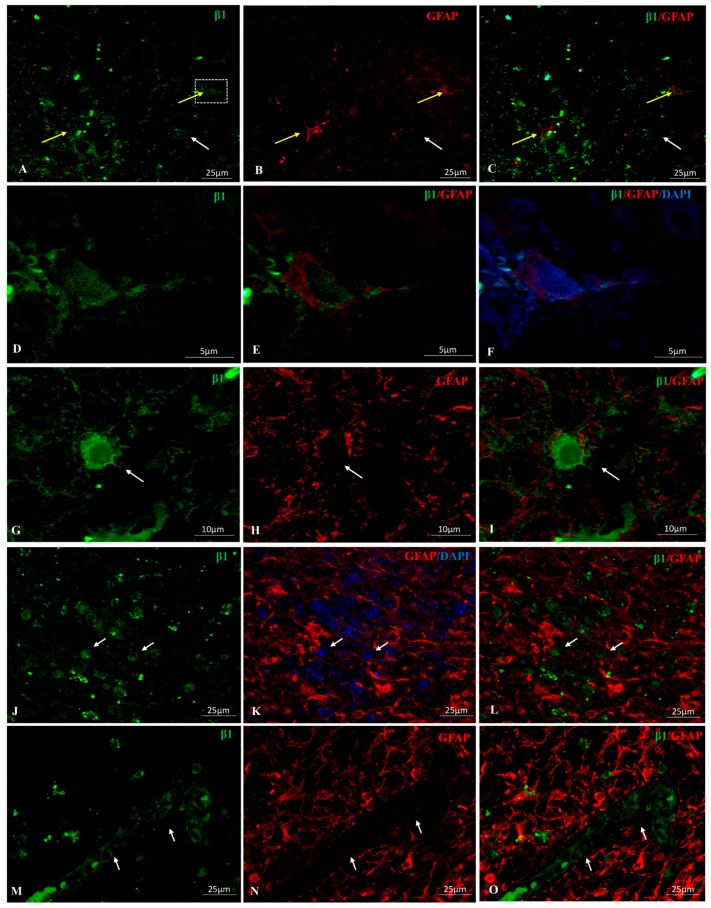
Double immunolocalization for GFAP (Glial Fibrillary Acidic Protein) (red) and Na-K-ATPase β1 subunit isoform (green) in primary (**A**–**I**) and secondary (**J**–**L**) glioblastoma multiforme (GBM). (**A**–**C**): Yellow arrows point to β1/GFAP positive cells. Faint β1 positive staining is observed in the nucleus of the cell located in the right side of the image (enlarged in panels (**D**–**F**)). White arrow points to a GFAP-cell expressing β1 in plasma membrane, nucleus and podosome-like structures. (**G**–**I**): β1+ immunostaining in cytoplasm, membrane and nuclear envelope of a giant cell. Arrow points to an invadosome β1+. The cell is filled by GFAP^+^ filaments. (**J**–**O**) Secondary GBM. β1 signal in the nuclear envelope and, sometimes, nucleosol of GFAP^−^ cells (arrows). Note the brighter fluorescence signal for GFAP in secondary over primary GBM. (**M**–**O**) Arrows point β1^+^ stromal and microenvironment cells, GFAP^−^.

**Figure 2:**
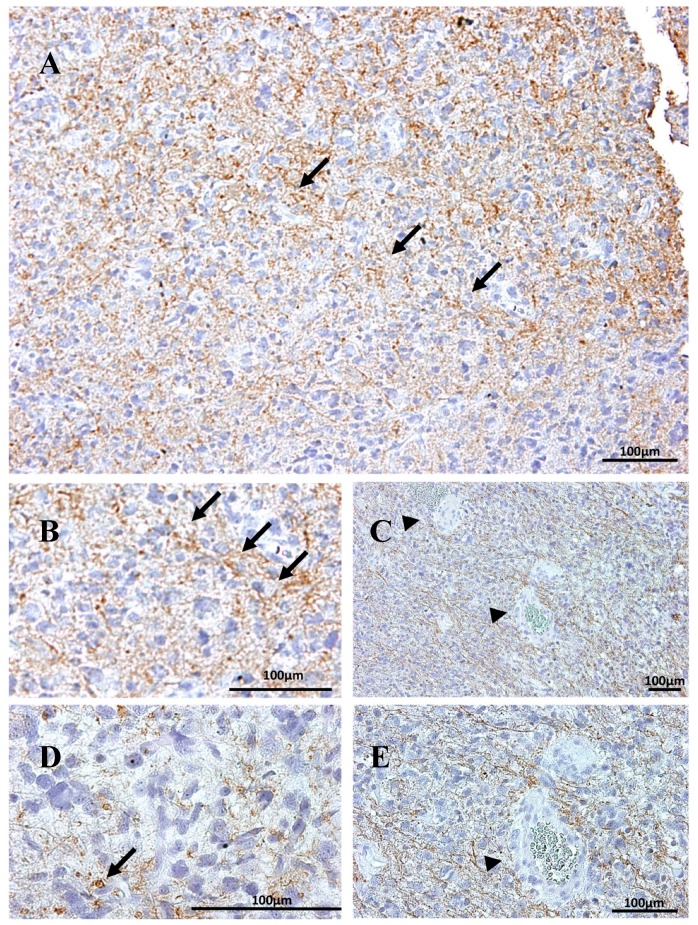
Immunoperoxidase staining for Na, K-ATPase β1 isoform in secondary GBM. (**A**) Stronger expression in the edge of the section becoming less intense inwards. Arrows point to the interface between the two zones. (**B**) Enlargement of the interface line. Na, K-ATPase β1^+^ cells appear mainly fibrillary (**B**,**C**,**E**), but also as rounded cells (arrow in (**D**)). (**C**,**E**) endothelial cells (arrowheads) are β1^−^ and vessels appear surrounded by β1^+^ fibers.

In GBM samples, the β1 isoform presented no clearly defined pattern of expression. This isoform was detected in some but not all tumor cells. The subcellular location differed among cells within a given area, while some tumor cells were immunopositive for β1 at the cytoplasmic membrane location (Figure 1A, white arrow) other cells presented immunostaining in a peri-nuclear localization (Figure 1G). Immunoperoxidase staining on paraffin-embedded tissue sections was used to localize Na, K-ATPase β1 isoform in samples from GBM patients. The immunoreactivity exhibited two distinct patterns: at the edge of the tumor there was strong positive and fibrillary staining (Figure 2A); β1 positive cells in the center of the section became less frequent and more globular (Figure 2D, arrows). The interface between the two zones was easily detectable (Figure 2A, arrows). In areas of blood vessel proliferation of, β1 positive pericyte-like cells were observed surrounding the vessels (Figure 1M), while endothelial cells varied from β1 negative to slightly positive (Figure 2C,D and Figure 5A, respectively).

In primary GBM, β1 expression in astrocytes was weak or absent (Figure 1A–C, yellow arrows and white arrow respectively). GFAP (Glial Fibrillary Acidic Protein) negative cells showed a variable β1-specific staining, mainly in plasma membrane and podosome/invadosome-like structures (Figure 1A–C,G–I, white arrows).

In secondary GBM, (Figure 1J–L), β1 immunoreactivity was predominantly located in the nuclear envelope, and sometimes, nucleosol of GFAP negative cells (arrows). Most of GFAP positive astrocytes did not show any β1-specific fluorescence signal. A morphologically heterogeneous pattern (multiform) was noted in different areas of the GBM sections, with areas where β1 isoform positive cells appeared mainly fibrillary (Figure 2), areas with β1-positive staining in the nuclear envelope of spherical cells (Figure 1J) and pericyte-like cells near blood vessels with a light β1-positive staining in the plasma membrane and the cytoplasm (Figure 1M).

### 2.2. Na, K-ATPase β2/AMOG Isoform Expression in GBM

**Figure 3:**
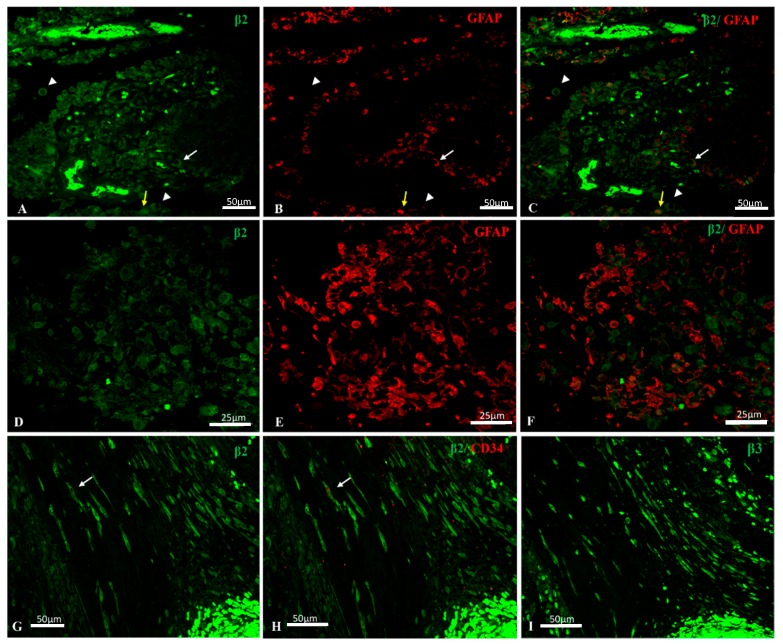
(**A**–**F**) Co-immunolocalization of Na, K-ATPase β2/AMOG (Adhesion Molecule On Glia) isoform (green) and GFAP (red) in GBM. (**A**–**C**) primary GBM. β2+ staining is mainly located in plasma membrane and less intense in cytoplasm of GFAP+ astrocytes. Some GFAP+ astrocytes show positive fluorescence in nuclei (yellow arrow) or in nuclear envelope (white arrow). In GFAP negative cells, β2/AMOG positive immunolocalization is present in cytoplasm and nuclear envelope (arrowheads). (**D**–**F**) In secondary GBM, Na, K-ATPase β2/AMOG isoform-specific labelling is of lower intensity than the observed in primary GBM and GFAP is more intense. (**G**,**H**) Positive immunostaining of β2/AMOG isoform in CD34+ (cluster of differentiation 34) telocyte-like cells (arrows). (**I**) CD34^+^/β2^+^ telocyte-like cells also express β3 isoform, mainly located in the cytosol (β2 is found in cytosol and more intense in plasma membrane and nuclear envelope).

In primary GBM, β2/AMOG signal was mainly located in the plasma membrane and in the cytoplasm at a lesser intensity in GFAP+ astrocytes; in some instances, positive fluorescence was observed in nuclei (Figure 3A–C, yellow arrow) or in nuclear envelope (Figure 3A–C, white arrow). Moreover, GFAP negative and β2/AMOG positive cells were observed (Figure 3A–C, arrowhead), although β2 expression was less intense than that observed in astrocytes and localized mainly in the cytoplasm and in the nuclear envelope. Definitively, β2/AMOG fluorescence signal in astrocytes was higher than that for β1.

In proliferating blood vessels, positive β2/AMOG staining was observed in endothelial cells and in other cells within the perivascular niche (Figure 3A).

In secondary GBM Na, K-ATPase β2/AMOG isoform-specific labelling was of lower intensity than that observed in primary GBM and GFAP was more intense (Figure 3D,E).

Co-immunolocalization of β2/AMOG and the telocyte marker CD34 (cluster of differentiation 34) was observed in some cells surrounding proliferating blood vessels (Figure 2G,H); these cells also expressed β3 (Figure 3, panel I).

### 2.3. Na, K-ATPase β3 Isoform Expression in GBM

**Figure 4:**
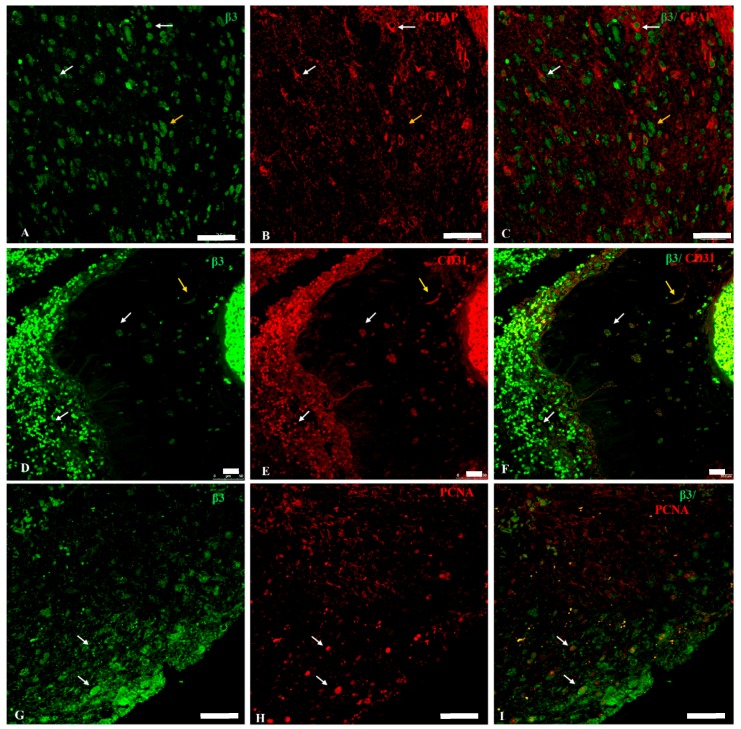
Double immunolocalization for GFAP (red) and Na, K-ATPase β3 subunit isoform (green) in primary and secondary GBM. (**A**–**C**) In primary GBM, most GFAP+ astrocytes show β3 positive labelling in the nucleus and nuclear envelope (white arrows). Yellow arrow points to a giant spindle-shaped cell β3 positive. (**C**) A+C merge. (**D**–**F**) Co-localization of β3 isoform and CD31 marker in secondary GBM shows positive β3 staining in cytosol and plasma membrane of CD31+ macrophages (white arrows) and in CD31+ pericytes (yellow arrow). (**G**–**I**) Co-localization of β3 isoform (green) and proliferating cell nuclear antigen (PCNA) (red). White arrows point to PCNA+ cells in which β3+ signal is observed in plasma membrane, cytoplasm and the nuclear envelope. Scale bar: 25 μm.

Most GFAP positive astrocytes exhibited β3 subunit positive immunolabelling (Figure 4A–C, white arrows) in the cytosol, nucleus and nuclear envelope. β3 positive labelling was also observed in giant, spherical or spindle-shaped cells (Figure 4A–C, yellow arrow).

**Figure 5:**
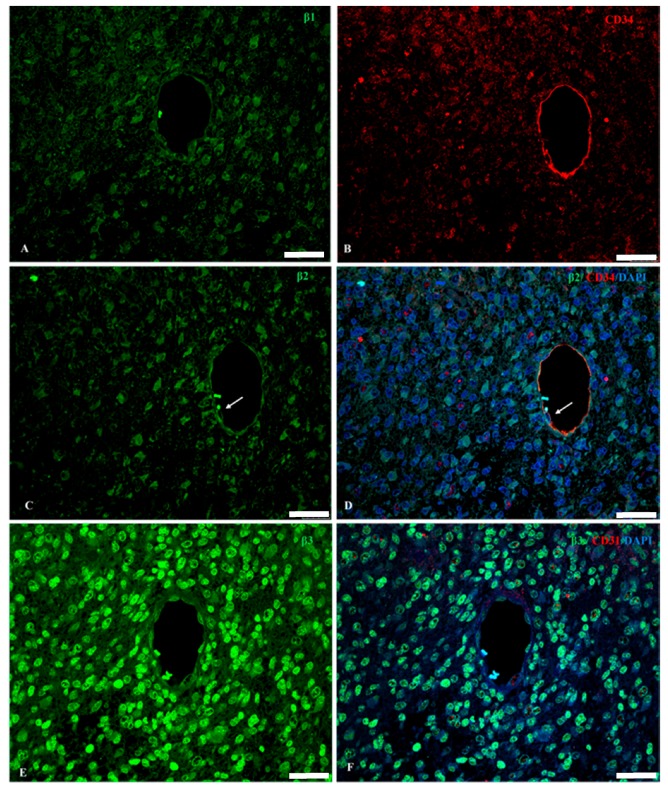
Serial sections from the same secondary GBM patient double immunostained for the 3 β-subunit isoforms (green) and the endothelial/macrophage/telocyte marker CD34 (cluster of differentiation) or the endothelial/monocyte-derived macrophage/pericyte marker CD31 (red). (**A**,**B**) light β1+ immunolabelling is present in the cytoplasm of CD34+ endothelial cells. (**C**) β2 positive staining in the cytoplasm and nuclear envelope (arrow) of endothelial cells. (**D**) β2, CD34 and DAPI merged image. (**E**) Nuclei of endothelial cells show a strong β3 immunostaining. Note the higher staining intensity for the β3 isoform in peripheral cells surrounding the blood vessel, compared to β1 and β2 immune labeling. (**F**) β3, CD31 and DAPI merged image. Scale bar: 40 μm.

Co-labelling with RNTβ3 and CD31 antibodies evidenced the expression of this isoform in CD31^+^ pericyte-like cells (Figure 4D,E, yellow arrow), in CD31^+^ macrophages (Figure 4D,E, white arrows) and in endothelial cells (Figure 5E).

PCNA^+^ (Proliferating Cell Nuclear Antigen) cells were β3 positive too, mainly in the nuclear envelope, in cytoplasm and in plasma membrane (Figure 4G–I, arrows). In determined areas of GBM samples, β3-specific fluorescence was brighter at the tumor front decreasing gradually to the interior (Figure 4G,I); thus, the ratio β3/PCNA in these areas was higher in the front and lower within the tumor (Figure 4G–I). In other areas, the β3 signal was homogeneous and very intense, both in the periphery and in the interior (Figure 5E).

### 2.4. Na, K-ATPase β-Isoforms Expression in Blood Vessel Cells and Perivascular Niche of GBM

Figure 5 shows serial sections from the same GBM patient double immunostained for the 3 β-subunit isoforms and the endothelial/macrophage/telocyte marker CD34 or the endothelial/monocyte-derived macrophage/pericyte marker CD31. β1 and β2 positive staining in endothelial cells was mainly located in the cytoplasm and nuclear envelope of endothelial cells (Figure 5A,C), while β3 was mainly located in the nucleus of such cells, with higher intensity compared to the other isoforms (Figure 5E). Moreover, a brighter fluorescence signal for the β3 isoform was observed in the peripheral cells surrounding the blood vessel compared to those of β1 and β2 immune staining (Figure 5).

## 3. Discussion

The physiological role of Na, K-ATPase in astrocytes is to remove the excess of K^+^ from the extracellular space after neuronal depolarization. However, transformed astrocytes in GBM harness ion channels and pumps, including Na, K-ATPase, to sustain their singular growth and invasion instead of regulation [58,59,60]. This study focuses on the Na, K-ATPase β subunit isoforms expression to determine their involvement in GBM oncobiology.

Table 2 summarizes the cell- and subcellular-specific Na, K-ATPase β subunit isoforms expression in primary and secondary GBM.

The three Na, K-ATPase β subunit isoforms (β1, β2, β3) were detected in both primary and secondary GBM. β1 expression was observed predominantly in the cell membrane and nucleus of GFAP negative cells, β2 in cytoplasm, plasma membrane and nuclei of astrocytes and β3 in the nuclei of astrocytes. In astrocytes of secondary GBM, β3 was also detected in cytosol and plasma membrane. Regarding expression levels, Na, K-ATPase β2 isoform expressed in astrocytes was lower in secondary GBM compared to primary GBM and β3 isoform expression was more intense in secondary GBM compared to primary GBM.

Na, K-ATPase β2 in a healthy brain is mainly expressed in astrocytes [61]. Conversely, other studies reported that in human gliomas β2/AMOG isoform was downregulated in neoplastic cells astrocytes, and this decrease in expression was correlated with increasing tumor grade and cell migration [56,57]. Moreover, we found that GFAP expression in secondary GBM was more intense than that observed in primary GBM, suggesting that astrocytes in primary GBM are less differentiated than those of secondary GBM. With this in mind, it would be logical to assume that primary GBM astrocytes present an equal or lesser expression of β2 than secondary GBM astrocytes, which has a slower progression. However, we found that in primary GBM the β2 isoform expression was more intense than that observed in secondary GBM (Table 2).

The Na, K-ATPase β1 isoform studied in mammal brains is predominantly expressed in neurons, and negligible in astrocytes [61]. We found in both primary and secondary GBM, that astrocytes did not express β1 or expressed it weakly; this is consistent with the findings in the literature referred to in the Introduction section. We also observed GFAP negative cells that expressed β1 subunit.

Regarding the oligodendrocyte-specific β3 isoform [40,61], we found positive expression in most GBM cells, either GFAP positive or negative. In secondary GBM there were more cells expressing β3 than in primary GBM, which may be due to a decrease in the β2 expression tied to an increase of β3 expression. The presence of the β3 isoform and the low expression of the β1 and β2 isoforms characteristic of astrocytes are consistent with an oligodendrocyte or oligodendrocyte progenitor phenotype [40].

Another important objective of this study was to correlate the mitotic index related to the expression of isoforms by co-localization of those along with PCNA, the clamp subunit of DNA polymerase δ marker of cell proliferation [62,63], and carry out further analysis by confocal microscopy. No correlation was seen between sodium pump isoforms and PCNA protein expression in GBM cells, that is, high expression of PCNA can be found in cells with either high or low expression levels of the different β isoforms, and vice versa.

Other than in gliomas, abnormal expression of Na, K-ATPase β subunit isoforms has been observed in many carcinomas. β1 and β2/AMOG mRNAs are decreased in renal, lung and hepatocellular carcinomas [64], and expression levels of the corresponding proteins are decreased in human clear cell renal cell carcinoma [51] and bladder carcinoma [65]. Previous work from our laboratory [66] reported opposite patterns of β1 isoform expression in gastric and colon adenocarcinomas in a recent study of subcellular expression of all α and β subunits isoforms in colorectal cancer [67]. The level of expression and the location of the β subunit in epithelial cells are important for maintaining their well-differentiated phenotype, which disappears during cancer progression. Further studies suggest that the transcription factor Snail might be repressing the β1 isoform and E-cadherin expression in carcinomas, associating these events to epithelial-mesenchymal transition (EMT) [65].

Variations in β isoforms expression patterns have been described in the regeneration of dorsal root ganglia and sciatic nerve [68], resembling to a certain extent, changes reported in GBM.

With the purpose of finding an β2/AMOG neuronal receptor-protein, the Matchmaker Two-Hybrid system from Clontech was used to pull-out β2/AMOG interacting factors [69,70]. “The two-hybrid assay is a sensitive in vivo method for identifying proteins that interact with protein of interest and is well-suited for detecting weak or transient interactions”. Full-length β2/AMOG prevented cell survival, thus, the protein was split into a carboxy-terminal fragment and an amino-terminal fragment and independently used for screening for interacting proteins in a Human Brain Matchmaker cDNA library (Clontech, Mountain View, CA, USA). 2.7 × 10^6^ clones were screened. Both carboxy and amino fragments allowed us to identify interacting proteins, including the cytosolic proteins, endoplasmic reticulum proteins and intra-nuclear proteins [71].

Invadosome formation is a key process in tumor progression including cell growth, angiogenesis, invasion and metastasis. A previous report from our laboratory noted the presence of podosome/invadopodia-like structures in the progression of GBM [72], showing that, with only one exception in the neurons, all described kinds of cells in GBM present podosome/invadopodia-like structures, including GBM-CSC (Cances Stem Cells) and tumor-associated macrophages (TAMs). Here we show evidence (Figure 1G–I and Figure 2D) of the presence of Na, K-ATPase β1 and β2 isoforms in podosome/invadopodia-like structures in the tumor invasion front, where GBM cells migrate towards the neighboring normal tissue by extending membrane protrusions (invadopodia) containing metalloproteinases (MMPs) [73]. However, a specific role for β isoforms in the invadosome of any non-α-associated β-isoform needs to be investigated.

**Figure 6:**
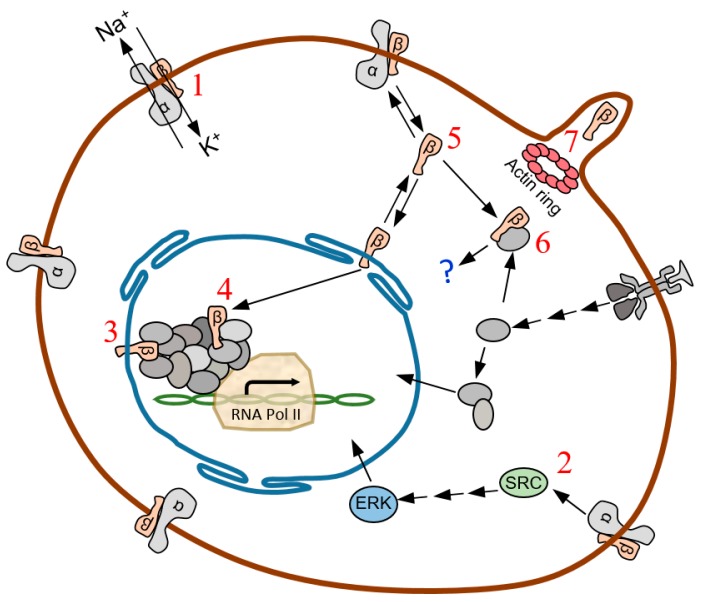
Na, K-ATPase β subunit isoforms functional fates. (1) as a subunit of the plasma membrane sodium pump αβ protomer [16]. (2) as a component of the cell de-differentiation and proliferation regulatory path Src-B-Raf-MEK-ERK [25]. (3–4) transcriptional regulator as co-activator of RNA polymerase II, embedded either in the nuclear envelope (3) or intranuclear (4). (5) in the cytosol, ready to be disposed to the plasma membrane or, (6) to act as an adaptor of a transduction signal canonical pathway or, (7) constituting a piece of the podosome/invadosome system.

Figure 6, as a graphical summary, shows the possible functional fate of Na, K-ATPase β subunit isoforms based on the results of this study and compared with current research. However, further studies need to be performed in order to precisely define the moonlighting roles of the Na, K-ATPase β isoforms as transcriptional co-activators or transduction signal adaptors and their potential use as biomarkers of specific GBM staging and progression.

## 4. Materials and Methods

### 4.1. Patients and Tumor Tissue

The study was approved by the Ethical Committee of Nuestra Señora de Candelaria University Hospital (HUNSC); Santa Cruz de Tenerife, Canary Islands, Spain (no. 198/2008, approved on 16 September 2008) and the Ethics Committee of La Laguna University (La Laguna, Canary Islands, Spain). All patients were treated in the HUNSC between years 2007 and 2017 and provided informed consent for the diagnosis and research of tissue specimens before entering the study. Clinical and pathology data were collected from 41 patients, 33 primary GBM (14 males and 19 females) and 8 secondary GBM (6 males and 2 female). GBM samples were taken after initial surgery before patients received radiation or chemotherapy. Paraffin-embedded tissue samples and corresponding clinical data were used ensuring patient’s anonymity.

### 4.2. Antibodies

Primary antibodies: rabbit polyclonal antibody SpETβ1 (anti-human-Na, K-ATPase β1 isoform) (dilution 1:600); rabbit polyclonal antibody SpETβ2 (anti-human-Na, K-ATPase β2 subunit isoform) (dilution 1:600) [74]; rabbit polyclonal antibody RNTβ3 (anti-Na, K-ATPase β3 subunit isoform) (dilution 1:100) [40]; mouse monoclonal antibody clone PC10 against anti-proliferating cell nuclear antigen (PCNA) (dilution, 1:100; #1486772 Roche Diagnostics GmbH, Mannheim, Germany); mouse monoclonal anti-human cluster of differentiation (CD)31 (ready-to-use; #IR610 Dako, Glostrup, Denmark); mouse monoclonal anti-Glial Fibrillar Acidic Protein (GFAP) (dilution 1:100; #G3896 Sigma, Saint Louis, MO, USA). Secondary antibodies: fluorescein isothiocyanate (FITC)-conjugated goat pAb against rabbit IgG (dilution 1:200; #F9887; Sigma-Aldrich, St. Louis, MO, USA); goat pAb against mouse IgG DyLight^®^ 650 (dilution 1:100; #ab97018; Abcam, Cambridge, UK).

### 4.3. Image Analysis and Statistical Analysis

Tables were compiled by two independent observers that evaluated the specimens blindly. Staining intensities were graded as strong (+++), moderate (++), weak (+) or absent (−). These cut-offs were established by consensus between each investigator. In the cases where the scores were different by more than one unit, the observers re-evaluated the specimens to reach a consensus. In other cases, the means of the scores were calculated.

### 4.4. Immunohistochemistry

Immunoperoxidase staining of 10% formalin-fixed paraffin-embedded tissue sections was performed using an ordinary avidin-biotin method. Briefly, after deparaffinization in xylene and hydration in a graded series of alcohol baths, tissue sections were heated in sodium citrate buffer (pH 6.0) at 120 °C for 10 min in an autoclave to achieve epitope retrieval. Non-specific sites were blocked with 5% non-fat dry milk in Tris buffered saline (TBS) for 1 h at room temperature. To block endogenous biotin, the Avidin/Biotin Blocking kit (#SP-2001, Vector Laboratories Inc., Burlingame, CA, USA) was used according to the manufacturer instruction. Primary antibodies were incubated over night at 4 °C. Endogenous peroxidase activity was blocked by incubating the slides with 3% hydrogen peroxidase in methanol for 15 min. Biotin-conjugated anti-rabbit secondary antibody was incubated for 2 h at 37 °C, and the specific antibody staining was amplified with the ABC Peroxidase Staining kit (Thermo Fisher Scientific, Inc., Waltham, MA, USA). 3,3′-diaminobenzidine substrate concentrate (#IHC-101F; Bethyl Laboratories Inc., Montgomery, TX, USA) was used to visualize immunohistochemical reactions. Samples incubated without primary antibodies were used as a negative control. Slides were counterstained with Harris hematoxylin solution DC (#253949, Panreac Química SLU, Barcelona, Spain) to visualize cell nuclei and mounted with Eukitt mounting medium (#253681, Panreac Química SLU, Barcelona, Spain). An optical light microscope (BX50; Olympus Corporation, Tokyo, Japan) was used to visualize the results of the immunostaining.

### 4.5. Double Immunofluorescence Simultaneous Staining

Immunofluorescent staining of 10% formalin-fixed paraffin-embedded tissue sections was performed as previously described [51]. Briefly, after deparaffinization in xylene and rehydrated in a graded series of alcohol baths, tissue sections were heated in sodium citrate buffer (pH 6.0) at 120 °C for 10 min in an autoclave to achieve epitope retrieval. Nonspecific sites were blocked with 5% bovine serum albumin or normal donkey serum in Tris-buffered saline (TBS) for 1 h at room temperature, tissue sections were then incubated simultaneously with a mixture of two distinct primary antibodies (i.e., rabbit against human target 1, mouse against human target 2) overnight at 4 °C. Slides were then incubated for 1 h at room temperature in the dark with a mixture of two secondary antibodies raised in different species and conjugated to different fluorochromes. Slides were mounted with ProLong^®^Diamond Anti-fade Mountant with DAPI (Molecular Probes^®^; Themo Fisher Scientific, Inc., Waltham, MA USA) to visualize cell nuclei. Slides were analyzed using Olympus FV1000 (Olympus Corporation, Tokyo, Japan) and Leica SP8 (Leica Microsystems, Wetzlar, Germany) confocal microscopes.

## 5. Conclusions

Assuming that β2/AMOG is involved in physiological astrocyte-neuron adhesion and the changes reported in this study on the quantitative and topological location of Na, K-ATPase β subunit isoforms, we also propose a role for these proteins in the egoist transformation of the astrocyte from its duty as neuron carer to GBM cancer cell.

## Figures and Tables

**Table 1:** Markers used in this study.

Antibody	Specificity
*SpETβ1*	Na, K-ATPase β1 subunit isoform
*SpETβ2*	Na, K-ATPase β2 subunit isoform
*RNTβ3*	Na, K-ATPase β3 subunit isoform
*Anti-GFAP*	Astrocytes
*Anti-PCNA*	Proliferative cells
*Anti-CD31*	Endothelial cells/Monocyte derived macrophages
*Anti-CD34*	Macrophages

**Table 2:** Cell- and subcellular-specific Na, K-ATPase β subunit isoforms expression in primary and secondary GBM.

**Primary GBM**	**β1**	**β2**	**β3**
Astrocytes	−/+ c,n	++ c,n,m	++ n
Endothelial cells	−/+ c	++ c,n	++ n
Pericytes	++ c,m	++ c,n	++ c,m
Telocytes	?	+++ c,n	+++ c
TAMs	++ m	++ c,n	++ c,m
Tumor cells	−/+ c,n,m	+ c,n,m	+++ c,n
**Secondary GBM**	**β1**	**β2**	**β3**
Astrocytes	−	−/+ c,n,m	+++ c,n,m
Endothelial cells	+ c,m	++ c,n	+++ n
Pericytes	+/++ c,m	?	++ c,m
Telocytes	−	+++ c,n	+++ c,n
TAMs	+ c,n,m	++ c,n	++ c,m
Tumor cells	−/+ c,n	+ c,m,n	+++ n

−, negative; +, low; +, moderate; +++, high; −/+, variable from low to absent; ++/+, variable from moderate to low; ?, indeterminate staining; m, membrane; c, cytoplasm; n, nucleus or nuclear envelope. TAMs: tumor associated macrophages.

## Abbreviations

ECM	Extracellular matrix
EMT	Epithelial-mesenchymal transition
FITC	Fluorescein isothiocyanate
DAPI	Diamond Anti-fade Mountant
GBM	Glioblastoma multiforme
GIMs	Glioma-infiltrating myeloid cells
Iba1	Ionized calcium binding adaptor molecule 1
IQGAP1	IQ motif containing GTPase-activating protein
MAP2	Microtubule-associated protein 2
PCNA	Proliferating cell nuclear antigen
TAMs	Tumor associated macrophages
TRITC	Tetramethylrhodamine B isothiocyanate
